# Imaging atherosclerosis in rheumatoid arthritis: evidence for increased prevalence, altered phenotype and a link between systemic and localised plaque inflammation

**DOI:** 10.1038/s41598-017-00989-w

**Published:** 2017-04-11

**Authors:** S. Skeoch, P. L. Hubbard Cristinacce, H. Williams, P. Pemberton, D. Xu, J. Sun, J. James, C. Yuan, T. Hatsukami, P. D. Hockings, M. Y. Alexander, J. C. Waterton, I. N. Bruce

**Affiliations:** 1 Arthritis Research UK Centre for Epidemiology, Centre for Musculoskeletal Research, Faculty of Medicine, Biology and Health, The University of Manchester, Manchester Academic Health Science Centre, Manchester, UK; 2 The Kellgren Centre for Rheumatology, NIHR Manchester Musculoskeletal Biomedical Research Unit, Central Manchester University Hospitals NHS Foundation Trust, Manchester Academic Health Science Centre, Manchester, UK; 3 Centre for Imaging Sciences, The University of Manchester, Manchester Academic Health Science Centre, Manchester, UK; 4 School of Psychological Sciences, The University of Manchester, Manchester, UK; 5 Department of Nuclear Medicine, Central Manchester University Hospitals NHS Foundation Trust, Manchester Academic Health Science Centre, Manchester, UK; 6 Specialist Assay Laboratory, Central Manchester University Hospitals NHS Foundation Trust, Manchester Academic Health Science Centre, Manchester, UK; 7 Department of Radiology, University of Washington, Seattle, USA; 8 MedTech West, Chalmers University of Technology, Gothenberg, Sweden; 9 Antaros Medical, Mölndal, Sweden; 10 Healthcare Science Research Institute, Manchester Metropolitan University, Manchester, UK

## Abstract

In rheumatoid arthritis (RA), chronic inflammation is thought to drive increased cardiovascular risk through accelerated atherosclerosis. It may also lead to a more high-risk plaque phenotype. We sought to investigate carotid plaque phenotype in RA patients using Dynamic Contrast-Enhanced MRI (DCE-MRI) and Fludeoxyglucose Positron Emission Tomography(FDG-PET). In this pilot study, RA patients and age/sex-matched controls were evaluated for cardiovascular risk factors and carotid plaque on ultrasound. Subjects with plaque >2 mm thick underwent DCE-MRI, and a subgroup of patients had FDG-PET. Comparison of MRI findings between groups and correlation between clinical, serological markers and imaging findings was undertaken. 130 patients and 62 controls were recruited. Plaque was more prevalent in the RA group (53.1% vs 37.0%, p = 0.038) and was independently associated with IL6 levels (HR[95%CI]: 2.03 [1.26, 3.26] per quartile). DCE-MRI data were available in 15 patients and 5 controls. Higher prevalence of plaque calcification was noted in RA, despite similar plaque size (73.3% vs 20%, p = 0.04). FDG-PET detected plaque inflammation in 12/13 patients scanned and degree of inflammation correlated with hs-CRP (r = 0.58, p = 0.04). This study confirms increased prevalence of atherosclerosis in RA and provides data to support the hypothesis that patients have a high-risk plaque phenotype.

## Introduction

Rheumatoid arthritis (RA) is a chronic autoimmune disease which affects 1% of the adult population^1^. It is characterised by synovial joint inflammation which causes progressive disabling arthritis. Patients also suffer with extra-articular disease and a high systemic inflammatory burden. RA is associated with premature death, the leading cause being cardiovascular disease (CVD)^2^. Studies have demonstrated that the excess burden of CVD in RA is equivalent to that seen in type two diabetes^3^. Evidence suggests a more aggressive clinical phenotype of CVD in RA, with patients having fewer warning symptoms prior to major events, and higher case-fatality rates^4, 5^.

Although an increased prevalence of some traditional risk factors is seen in RA, they do not fully explain the excessive risk. Chronic inflammation is thought to be a major driver of CVD in this population^6^. Although mechanisms are unclear, inflammation may not only influence traditional cardiovascular risk factors such as dyslipidaemia, but also have direct detrimental effects on the vessel wall^7, 8^. Recent therapeutic advances in RA have led to significant improvements in outcomes of joint disease. However, the mortality gap between those with and without RA persists, and excess cardiovascular risk is still evident^9, 10^. A better understanding of the underlying pathological processes driving increased risk, and the potential effects of RA treatment on vascular pathology, would improve our ability to target cardiovascular risk reduction more effectively in this population.

Atherosclerosis, the pathological mechanism underlying most CVD in RA, is also a chronic inflammatory condition with immune cells playing a key role in all stages of plaque development^11^. In addition, plaque rupture (the main trigger for clinical events) is influenced by plaque composition and presence of inflammation rather than simply by plaque size^12, 13^. High risk plaque features include the presence of calcification, lipid rich necrotic core (LRNC), neovascularisation and inflammatory cell infiltration. In the general population there is evidence linking pro-inflammatory cytokines with plaque progression and destabilisation^14^. IL6 and TNF levels have been shown to be independent predictors of future cardiovascular mortality in large meta-analyses^15, 16^. In RA, an increased prevalence and faster progression of atherosclerosis is associated with inflammation^17, 18^ and there is emerging evidence that RA patients may have a more rupture-prone plaque phenotype^19^. In a post mortem study, Aubrey *et al*. demonstrated less stenosed, more inflammatory coronary plaques in RA patients who had died from acute myocardial infarction (MI)^19^. Furthermore, Karpouzas *et al*., found that RA patients had a higher prevalence of high risk plaques on coronary CT^20^. The clinical phenotype of CVD with more sudden severe events may partly be explained by the hypothesis that patients have a more rupture prone phenotype^5, 21^.

Recent advances in non-invasive carotid artery imaging allow more detailed characterisation of plaque phenotype. Carotid plaque rupture is an important cause of stroke, however presence and composition of carotid plaque are also predictors of coronary disease^22^. Carotid magnetic resonance imaging (MRI) can be used to measure plaque burden and compositional features including LRNC, calcification and fibrous cap integrity^23^. A strong correlation has been established between MRI and histological findings, and MRI has been used in clinical studies to evaluate predictors of stroke, and response to statin therapy^24, 25^. More recently, carotid artery dynamic contrast enhanced MRI (DCE-MRI) has been developed to measure plaque neovascularisation and inflammation in stroke patients^26^. Serial images are taken before, during and after contrast injection, then contrast kinetics are modelled to estimate the transfer constant (*K* ^trans^) of contrast and the fractional volume of plasma within the plaque (*v*_P_). Both *K* ^trans^ and *v*_P_ have been shown to correlate with microvessel density, while *K* ^trans^ also correlated with macrophage infiltration of the plaque on histology^27^.

^18^F-fludeoxyglucose positron emission tomography (FDG-PET) is a nuclear imaging technique which can also be used to quantify carotid plaque inflammation. FDG is taken up preferentially in metabolically active tissues and when combined with MRI or CT, activity can be mapped to an anatomical location. In stroke patients, FDG is taken up preferentially in ‘culprit’ lesions and uptake associates strongly with macrophage density on histology^28^.

Maki-Petaja demonstrated an improvement in aortic inflammation on FDG-PET-CT in RA patients treated with anti-TNF therapy^8^. Haavisto *et al*. also recently demonstrated that carotid artery inflammation improved following treatment with anti-inflammatory therapy^29^. Although FDG-PET has been used to characterise vascular inflammation in RA, neither MRI nor PET have been utilised to investigate atherosclerotic plaque phenotype in RA. However the combination of imaging techniques could provide a comprehensive method with which to interrogate the relationship between chronic inflammation and atherosclerosis in RA, thus allowing a more stratified approach to CVD risk management.

The aim of the current study was test the hypothesis that RA patients have a high-risk carotid plaque phenotype which can be characterised non-invasively using DCE-MRI and PET-CT. We also aimed to evaluate the association between clinical and serological markers of RA disease activity and plaque presence and characteristics. Finally we aimed to test the feasibility of utilising DCE-MRI and FDG-PET to study carotid atherosclerosis in an RA population.

## Patients and Methods

### Study population

A cross-sectional pilot study of patients with established active RA (defined as a disease activity 28 score (DAS28) >3.2, diagnosed for more than 1 year) who fulfilled the 1987 ACR Criteria for RA^30^, together with age and sex matched controls, was undertaken. Patients were recruited from outpatient clinics in the North West of England between September 2012 and October 2014. Controls were recruited through the “best friend” method, whereby participants invite a friend to consider participation. Additionally, controls were recruited via advertisements within The University of Manchester and mailshots through The Greater Manchester Primary Care Research Network. Participants aged between 18 and 70 were included. Major exclusion criteria included recent statin use (within 2 months of inclusion), significant renal impairment (estimated glomerular filtration rate <50 ml/min/1.73 m^2^), any contra-indication to MRI, and a history of vasculitis. The study was approved by the NHS Health Research Authority (North West Research Ethics Committee, reference: 12/NW/0117). All participants provided written informed consent and all methods were performed in accordance with the ethically approved protocol.

### Data collection

#### Clinical and laboratory assessments

Participants underwent assessment of traditional cardiovascular risk factors and, in patients, RA disease characteristics were assessed. This included assessment of joint disease activity using the DAS28 score: a composite measure of tender and swollen joints, patient reported disease activity and serological measure of inflammation which has been widely validated and is used in routine clinical practice. Disability was also evaluated using The Stanford Health Assessment Questionnaire (HAQ) in addition to history of extra-articular disease and treatment history. Fasting bloods were drawn for lipids, glucose, renal function, ESR measurement. High-sensitivity C-reactive protein (hs-CRP) was measured by an in-house ELISA method using anti-human CRP antibodies, calibrators and controls from Abcam (Cambridge, UK). Serum IL6, ICAM, VCAM1, E-selectin and P-selectin levels were all measured by ELISA using DuoSet development kits from R&D Systems (Abingdon, UK).

#### Carotid artery ultrasound

An ultrasound (US) was performed to screen for carotid plaque in the carotid bulb, common carotid artery and internal carotid artery within 2 cm above and below the bifurcation by one of three trained sonographers. All scans were performed on a Philips iU22 machine using a 9–3 MHz probe and standardised vascular protocol settings. Both arteries were scanned in transverse and longitudinal planes with the participants in a supine position. Plaque was defined in accordance with published recommendations (2 out of 3: Intimal medial thickening >1.5 mm, increased echogenicity, luminal protrusion^31^). For carotid plaque assessment on MRI, a minimum plaque thickness of >2 mm is required. Therefore only participants with plaques >2 mm thick were invited for MRI.

#### MRI assessment

Suitable participants attended for MRI within 2 weeks of initial assessment. Scans were performed on a 3T Philips Achieva MRI scanner (Philips Healthcare, The Netherlands) with a specially designed 8-channel carotid artery coil (SHCG, China). A number of sequences were acquired including T_1_-weighted, T_2_-weighted, proton density, magnetisation prepared rapid acquisition gradient echo (MP-RAGE) and time of flight angiography sequences (protocol details in supplemental data).

A DCE sequence was then performed. Briefly, 4 slices were positioned around the plaque and 20 frames were acquired (TR:126 ms, TE:4.61 ms, flip angle 50°, slice thickness 3 mm, matrix 260 × 260). 2 frames were acquired prior to injection of gadopentate dimeglumine (Magnevist, Bayer HealthCare Pharmaceuticals), concentration 0.05 mmol/kg, injection rate: 1cc/sec then 18 frames acquired during and after injection (17.5 s/frame).

Scans were anonymised and transferred to the Vascular Imaging Laboratory, University of Washington then analysed with CASCADE software using previously published methods (CASCADE, Seattle, WA)^32, 33^. Scans were analysed by 2 blinded readers. Plaque dimensions were measured and a normalised wall index (a measure of plaque burden) was calculated by dividing the measured wall area by the sum of the wall and luminal area in each slice. Then compositional features were measured including presence and size of LRNC, calcification, intra-plaque haemorrhage and fibrous cap integrity. Contrast kinetics within the plaque were evaluated using a 2-compartmental Patlak model from which *K* ^trans^ and *v*_P_ were estimated.

#### FDG-PET sub-study

Consecutive RA patients undergoing MRI who had no history of cancer, uncontrolled diabetes or infection were invited to have a carotid FDG-PET-CT scan. Scans were performed on a Siemens Biograph mCT·64·S (Siemens Healthcare, Germany). Patients fasted for 6 hours prior to intravenous injection of 200MBq ^18^F-FDG. Following a 2 hour resting/uptake period the PET-CT scan was performed, localised around the carotid bifurcation of the index artery (detailed methods in supplemental data).

**Figure 1:**
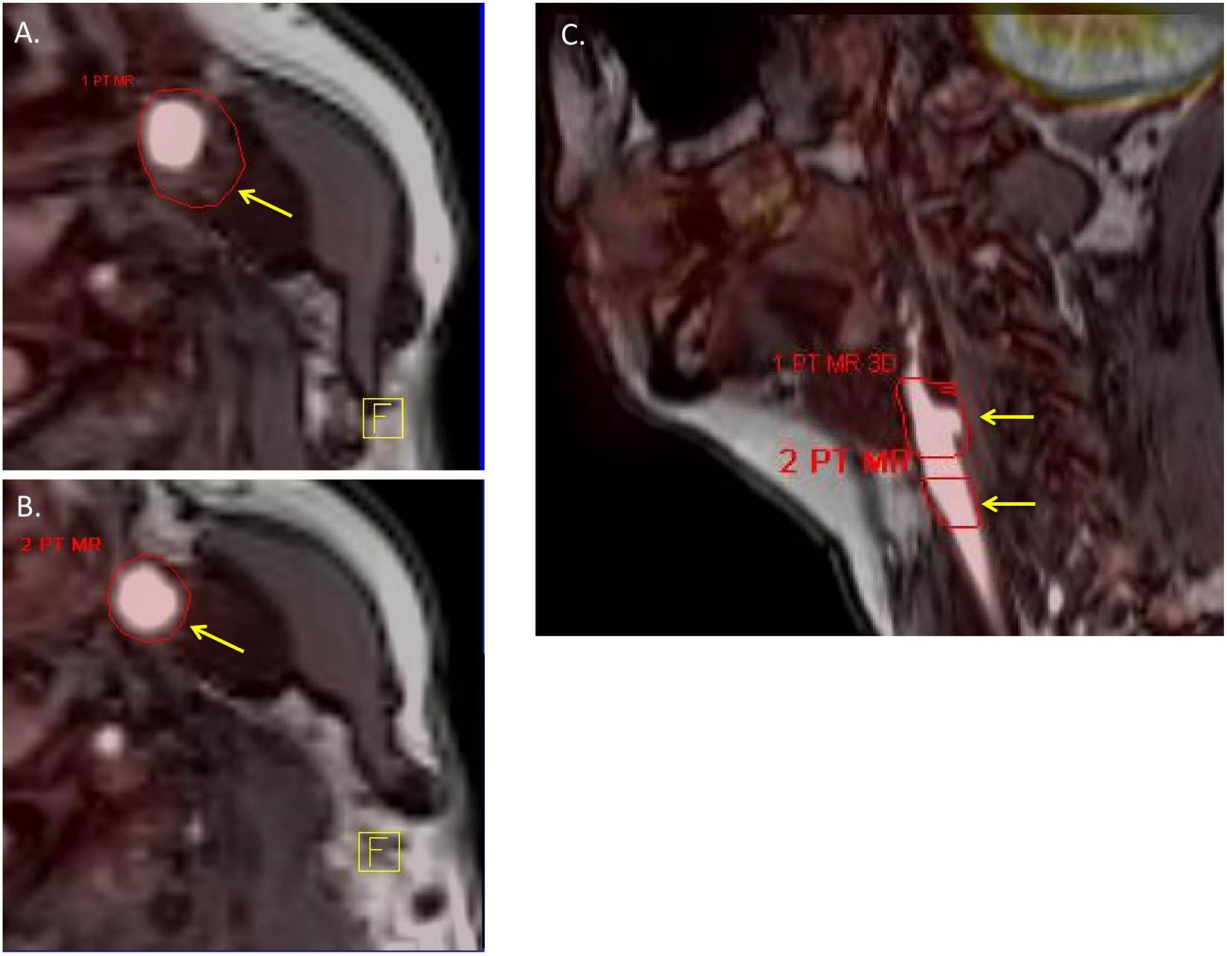
Identification of VOIs on PET-MRI. ROIs were drawn around the borders of plaque on each slice where plaque was seen (an example is seen in the axial image (**A**), yellow arrow points to VOI border). An equivalent number of ROIs are drawn around vessel wall on slices where no plaques are seen (an example is seen in (**B**)). An example of the ROIs on a sagittal section can be seen in (**C**) where each arrow points to the two defined ROIs, the superior one includes the atheromatous wall while the inferior ROI is of non-atheromatous wall.

PET-CT images were reconstructed using Siemens UHD (see supplemental data) then co-registered with MRI T_1_-weighted images. Volumes of interest (VOIs) were drawn around the affected artery on MRI, then plaque FDG uptake measured as the maximum standardised uptake values (SUV_max_). A second VOI was used to derive SUV_max_ from non-atheromatous wall. An example of VOIs on PET-MRI can be seen in Fig. 1. The physicist undertaking the analysis was blinded to clinical, serological and MRI measurements.

### Statistical analysis

This was a pilot study therefore no formal sample size was undertaken. Data were non-normally distributed thus cohort characteristics were summarized using median and interquartile range(IQR). Descriptive statistics were undertaken to compare clinical, serological and imaging findings in patient and control groups using non-parametric statistics. Associations of CV risk factors and RA related factors with presence of plaque were evaluated. Discrete variables were split into quartiles for further analysis. Hs-CRP and IL6 levels were undetectable in some subjects, therefore results were split into quartiles for analysis of association with plaque presence. Univariable logistic regression was used to evaluate associations of plaque with traditional and RA related risk factors. The relationship of RA related factors with plaque was interrogated with logistic regression models with age adjustment and then with adjustment for traditional risk factors. Finally a backwards stepwise multivariable regression was used to identify factors independently associated with plaque in RA patients. Association of disease activity and risk markers with localised plaque inflammation measured on MRI and PET were evaluated using the Spearman rank test.

## Results

**Figure 2:**
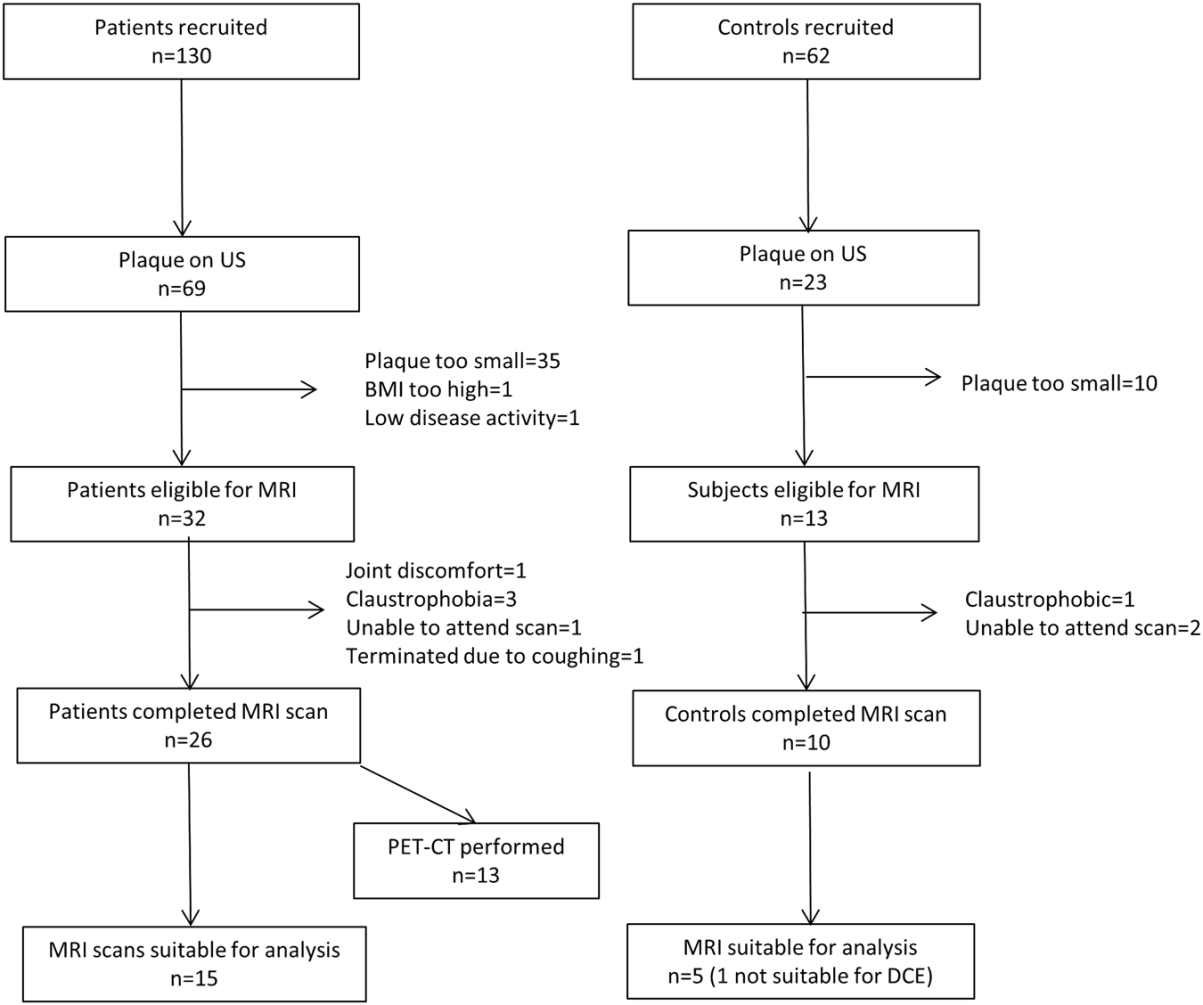
Schematic flow of data collection in patients and controls recruited into the study.

130 patients and 62 controls were recruited. Figure 2 demonstrates the flow of participants through the study. Baseline characteristics are summarised in Table 1. There were no significant differences in age or gender between the groups. However there was a higher prevalence of hypertension in the patient group (17.7% vs 3.9%, *p* = 0.01), and a higher systolic blood pressure was also noted compared with controls (136 vs 127 mmHg, *p* = 0.009). Patients also had lower total cholesterol levels and lower high density lipoprotein cholesterol (*p* = 0.013 and *p* = 0.045, respectively). There was an increased prevalence of carotid plaque in patients compared to controls (53.1% vs 37.0%, *p* = 0.038). When logistic regression was used to adjust for traditional cardiovascular risk factors and history of clinical CVD, the association between RA diagnosis and presence of plaque remained significant (odds ratio [95%CI]: 2.50 [1.08, 5.79]). Factors associated with presence of carotid plaque in patients are summarised in Table 2.

**Table 1:** Cohort Characteristics.

Variable	Patients (n = 130)	Controls (n = 62)	p-value
Traditional risk factors			
Sex (f)	99 (76.15)	48 (82.76)	0.311
Age (years)	55.44 (48.83, 61.87)	56.53 (50.38, 59.33)	0.957
Prior CVE	2 (1.54)	1 (1.67)	0.878
Smoker	18 (13.85)	4 (6.45)	0.133
TC:HDL	3.22 (2.68, 4.13)	3.28 (2.69, 3.87)	0.515
TC (mmol/l)	5.1 (4.6, 5.8)	5.5 (5.0, 6.1)	0.013
LDL (mmol/l)	2.96 (2.52, 3.6)	3.27 (2.68, 3.82)	0.068
HDL (mmol/l)	1.6 (2.82, 1.96)	1.71 (1.42, 2.06)	0.047
Diabetes	2 (1.54)	1 (1.67)	0.861
Fasting glucose (mmol/l)	4.9 (4.6, 5.3)	5.2 (4.8, 5.5)	0.016
Hypertension	23 (17.69)	2 (3.85)	0.0142
Systolic BP (mmHg)	136 (124, 148)	127 (117, 142)	0.009
Diastolic BP (mmHg)	81 (76, 89)	78.5 (74, 86)	0.213
Family history of CVE	43 (33.08)	17 (28.33)	0.497
Non classical risk markers			
hs-CRP (mg/l)	2.89 (1.01, 6.18)	0.79 (0.35, 2.05)	<0.001
TNF (pg/ml)	1 (1, 45.3)	1 (1, 1)	0.125
IL-6 (pg/ml)	1.99 (0.25, 4.69)	0.52 (0.25, 1.46)	<0.001
I-CAM (ng/ml)	146.7 [117.5, 186.1]	121.7 [106.9, 154.1]	0.004
V-CAM1 (ng/ml)	368.5 (314.6, 449.2)	375.2 (327.7, 435.45)	0.748
E-selectin (ng/ml)	7.24 (4.18, 15.89)	4.51 (1.37, 6.50)	0.001
P-selectin (ng/ml)	32.41 (25.71, 39.92)	29.69 (24.27, 36.29)	0.140
Carotid plaque	69 (53.08)	23 (37.01)	0.038
RA disease characteristics			
Disease duration	10.2 (5.3, 20.9)	—	—
DAS-28 score	4.62 (3.76, 5.47)	—	—
HAQ score	1.36 (0.50, 2.13)	—	—
RF positive	97 (74.62)	—	—
ACPA positive	107 (82.31)	—	—
Seropositive (ACPA+/or RF)	114 (87.69)	—	—
Extra-articular disease	35 (26.9)	—	—
Current methotrexate use	80 (61.5)	—	—
Any DMARD therapy (including biologics)	115 (88.5)	—	—
Current biologic therapy	38 (29.2)	—	—
Current oral glucocorticoids	15 (11.5)	—	—

Median (IQR) or Frequency (%) where variables are categorical. CVE: cardiovascular event, TC: total cholesterol, LDL: low density lipoprotein, HDL: high density lipoprotein, ACPA: anti citrullinated peptide antibody, RF: rheumatoid factor, DAS28: disease activity 28 score, HAQ: health assessment questionnaire, hs-CRP: high sensitivity C-reactive protein, IL-6: interleukin 6, I-CAM: intercellular adhesion molecule, VCAM-1: vascular cellular adhesion molecule 1.

**Table 2:** Factors associated with presence of carotid plaque in patients.

Predictor	Unadjusted OR (CI 95%)	OR(CI95%) with age adjustment	OR (95%CI) with adjustment for traditional Risk factors^Δ^	OR (95%CI) in fully adjusted model†
Age (years)	1.10 (1.04, 1.15)	—	—	1.10 (1.04, 1.18)
Gender (Male)	0.93 (0.41, 2.07)	0.89 (0.36, 2.18)	—	—
Current smoker	2.60 (0.87, 7.77)	4.34 (1.30, 14.45)	—	8.523 (0.872, 88.13)
Hypertension	1.47 (0.58, 3.69)	1.02 (0.38, 2.73)	—	—
TC:HDL	1.11 (0.85, 1.44)	1.15 (0.86, 1.55)	—	—
Glucose	1.06 (0.79, 1.44)	1.07 (0.80, 1.44)	—	
Disease duration (years)	1.02 (0.99, 1.06)	1.02 (0.97, 1.05)	—	—
DAS28	1.66 (1.18, 2.31)	1.38 (1.02, 1.89)	1.5 (1.059, 2.35)	—
HAQ*	1.63 (1.11, 2.41)	1.50 (1.00, 2.25)	1.45 (0.90, 2.35)	—
Extra-articular disease	0.92 (0.45, 1.90)	0.81 (0.371, 1.75)	0.72 (0.27, 1.89)	—
Seropositivity	1.17 (0.41, 3.34)	1.17 (0.55, 5.3)	0.80 (0.213, 3.06)	—
Current biologic therapy	1.06 (0.49, 2.27)	1.52 (0.649, 2.268)	1.41 (0.554, 2.58)	—
Traditional DMARD therapy	0.792 (0.341, 1.84)	0.690 (0.32, 1.48)	0.792 (0.341, 1.84)	—
Current oral glucocorticoid therapy	0.993 (0.337, 2.92)	0.991 (0.310, 3.17)	0.884 (0.249, 3.13)	—
hsCRP *	1.52 (1.10, 2.12)	1.41 (1.00, 1.99)	1.40 (0.96, 2.06)	—
IL-6 *	1.45 (1.06, 1.98)	1.40 (1.01, 1.96)	1.55 (1.07, 2.25)	2.03 (1.26, 3.26)
VCAM-1	1.004 (1.001, 1.007)	1.003 (1.00, 1.007)	1.002 (0.99, 1.006)	—
ICAM	1.007 (1.002, 1.012)	1.007 (1.001, 1.01)	1.004 (0.997, 1.010)	—
E-selectin	1.020 (1.0, 1.041)	1.022 (0.998, 1.045)	1.01 (0.99, 1.03)	—
P-selectin	0.996 (0.970, 1.02)	0.987 (0.959, 1.01)	0.99 (0.96, 1.03)	—

Quartiles where*. ^Δ^Adjusted for age, gender, hypertension, smoking, TC:HDL, glucose. ^†^Stepwise logistic regression with significance level set at <0.1. Variables included in model: age, gender, hypertension, smoking, TC:HDL, DAS28, HAQ, hs-CRP, IL-6, biologic therapy traditional DMARd therapy and current oral glucocorticoid therapy. Traditional DMARD therapy includes: methotrexate, sulfasalazine, hydroxychloroquine, leflunomide, gold.

On univariable analysis age and smoking status were the only traditional risk factors associated with plaque. However there were also significant associations with RA related factors: DAS28 score, disability, ESR, IL6 and hs-CRP levels (as shown in Table 2). On adjustment for traditional risk factors, the association of plaque with DAS28 score and IL6 remained significant (detailed in Table 2). On multivariable analysis IL6 levels, age and smoking remained in the model (OR [95%CI]: 2.03 [1.26, 3.26], 1.10 [1.04, 1.18] and 8.523 [0.872, 88.13], respectively). While IL6 and hs-CRP were significantly correlated (spearman rho = 0.38, p < 0.001), re-testing of the stepwise regression without hs-CRP did not lead to any change in the point estimate for IL6. Additionally removal of IL6 from the regression did not lead to inclusion of hs-CRP in the final model, suggesting that co-linearity was not a major issue in the analysis.

Circulating markers of endothelial activation: ICAM and e-selectin were increased in patients compared with controls (*p* = 0.04, *p* < 0.001, respectively) and were associated with presence of plaque on univariable analysis (OR [95%]: 1.007 [1.002, 1.012], 1.020 [1.0, 1.041], respectively). While VCAM-1 levels were not significantly higher in patients, they were associated with presence of plaque (OR [CI95%]: 1.004 [1.001, 1.007]).

### Plaque phenotype and inflammation on MRI

**Figure 3:**
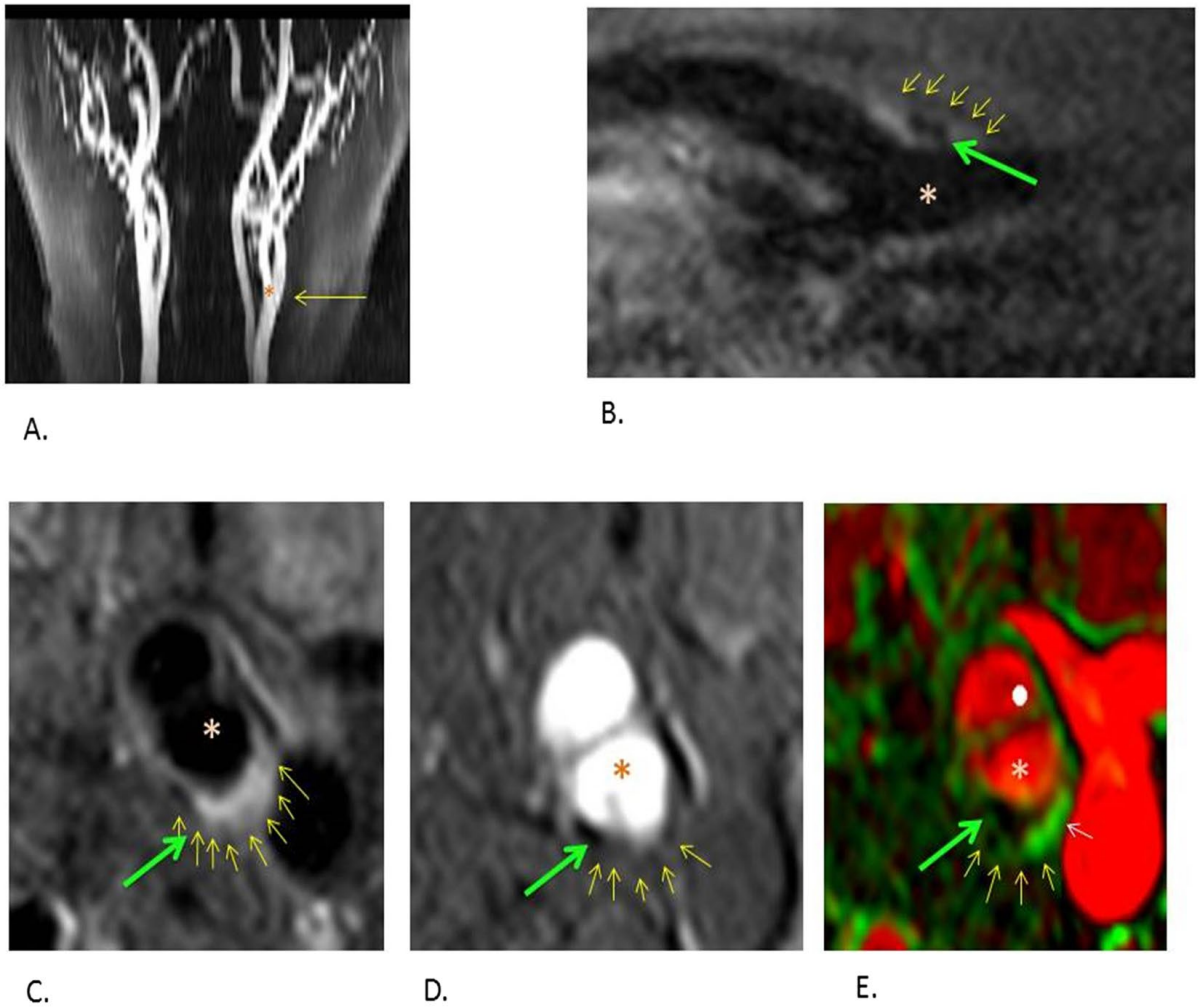
Representative images acquired during MRI. The small yellow arrows correspond to the outer border of plaque, the green arrows highlight an area of calcification within plaque and *highlights the lumen in each sequence. (**A**) A 2D time of flight with the yellow arrow pointing towards the bifurcation. (**B**) A black blood sagittal oblique section through the bifurcation. (**C**) A corresponding cross sectional black blood imaging sequence through the common carotid artery bifurcation where plaque can be seen at the posterior aspect of the vessel wall. (**D**) A corresponding bright blood image at the same level. (**E**) A DCE parameter map of the cross sectional image. Ktrans is estimated using a Patlak model and a Ktrans map is generated. The Ktrans signal is shown in green. The boundaries of the plaque defined on the T1 weighted sequence are applied to this map to estimate the Ktrans measurement within the plaque. The white arrow highlights the Ktrans signal within the plaque area.

As shown in Fig. 2, of the plaques identified on US, 35/69 in patients and 8/20 in controls did not meet suitability criteria for MR scanning. Additionally, although the scans were generally well tolerated by subjects, a number of MR scans were aborted in both groups (n = 4) and 16/36 scans were not suitable for analysis. In most cases despite satisfactory imaging quality plaques were not large enough for DCE analysis. Analysis was completed in 15 patients and 5 controls (DCE analysis was not possible in one control due to slice positioning). A representative MR image of plaque is shown in Fig. 3.

The RA group undergoing MRI were older than the whole RA cohort (59.6[56.2, 64.5] vs 54.2[48.0, 60.9] years, *p* = 0.004) and had higher median ESR (29.5[17, 46] mm/hr *vs* 13[7, 27] mm/hr, *p* = 0.002). There were no significant differences in any other traditional cardiovascular risk factors or disease related factors among the RA group with MRI data and the whole RA cohort.

The prevalence of traditional risk factors in the patients and controls in whom MRI data were analysed, is described in Table 3. There was no difference in age (median [IQR]: 59.6[56.2, 64.9] and 59.0[52.5, 65.5] years, p = 0.57). Two patients but no controls were smokers. There was a trend towards higher LDL levels in the control group (median [IQR]: 2.96[2.13, 2.28] and 3.87[3.28, 4.33] mmol/l in patients and controls respectively, p = 0.081) and higher systolic blood pressure in the patient group (median [IQR]: 147[129, 161] vs 127[117, 140] mmHg in patients and controls respectively, *p* = 0.079). One subject in the RA group had a history of stable angina. No control subjects had a history of clinical CVD.

**Table 3:** Differences in plaque characteristics on MRI and traditional risk factors between the groups. Median (IQR) or frequency (%) where*.

Characteristics	Patient	Control	P value
Plaque characteristics			
Plaque volume (mm^3^)	351.64 (217.14, 453.12)	309.93 (149.93, 706.12)	0.813
*K* ^trans^ plaque (min^−1^)	0.0448 (0.030, 0.785)	0.0822 (0.569, 0.104)	0.194
*v*_p_ plaque (0–1)	0.070 (0.050, 0.097)	0.0478 (0.043, 0.274)	0.484
Remodelling index	0.510 (0.480,0.60)	0.60 (0.485, 0.645)	0.483
Calcium present*	11 (73.33)	1 (20.00)	0.038
LRNC present*	13 (86.67)	4 (80.00)	0.560
LRNC volume (mm^3^)	29.12 (15.20, 49.16)	61.26 (25.28, 95.38)	0.358
Traditional risk factors			
Age (years)	59.6 (56.2, 64.9)	59.0 (52.5, 65.5)	0.57
Current smoker	2 (13.3)	0	—
LDL (mmol/L)	2.96 (2.13, 2.28)	3.87 (3.28, 4.33)	0.081
HDL (mmol/L)	1.55 (1.17, 2.01)	1.33 (1.25, 1.72)	0.860
TC:HDL	3.33 (2.48, 4.04)	4.36 (3.77, 4.87)	0.176
Systolic BP (mmHg)	147 (129, 161)	127 (117, 140)	0.079
Diastolic BP (mmHg)	87 (79, 89)	78 (71, 89)	0.342
History of hypertension	1 (6.67)	0	
History of diabetes	0	0	
History of clinical CVD	1 (6.67)	0	

*K* ^trans^: transfer constant, *v*_p_: partial volume of plasma, LRNC: lipid rich necrotic core, LDL low density lipoprotein, HDL: high density lipoprotein, BP: blood pressure.

There was no significant difference in plaque size between the groups (seen in Table 3). However, there was a significantly higher prevalence of calcification in patients compared to controls (73.3% vs 20%, *p* = 0.038). There were no significant differences in any other compositional features. While a degree of contrast enhancement was demonstrated in all patients, there was no significant difference in DCE parameters between the two groups. There was a trend towards an inverse association between calcium content of plaque and *K* ^trans^ measurement in patients (r-0.448, *p* = 0.093). There was no significant association between *K* ^trans^ measurements in plaque and hs-CRP levels (r = 0.002, *p* = 0.99), IL6 levels (r = 0.26, *p* = 0.36) or DAS28 score (r = −0.125, *p* = 0.61).

### Plaque inflammation on ^18F^FDG-PET-MRI

**Figure 4:**
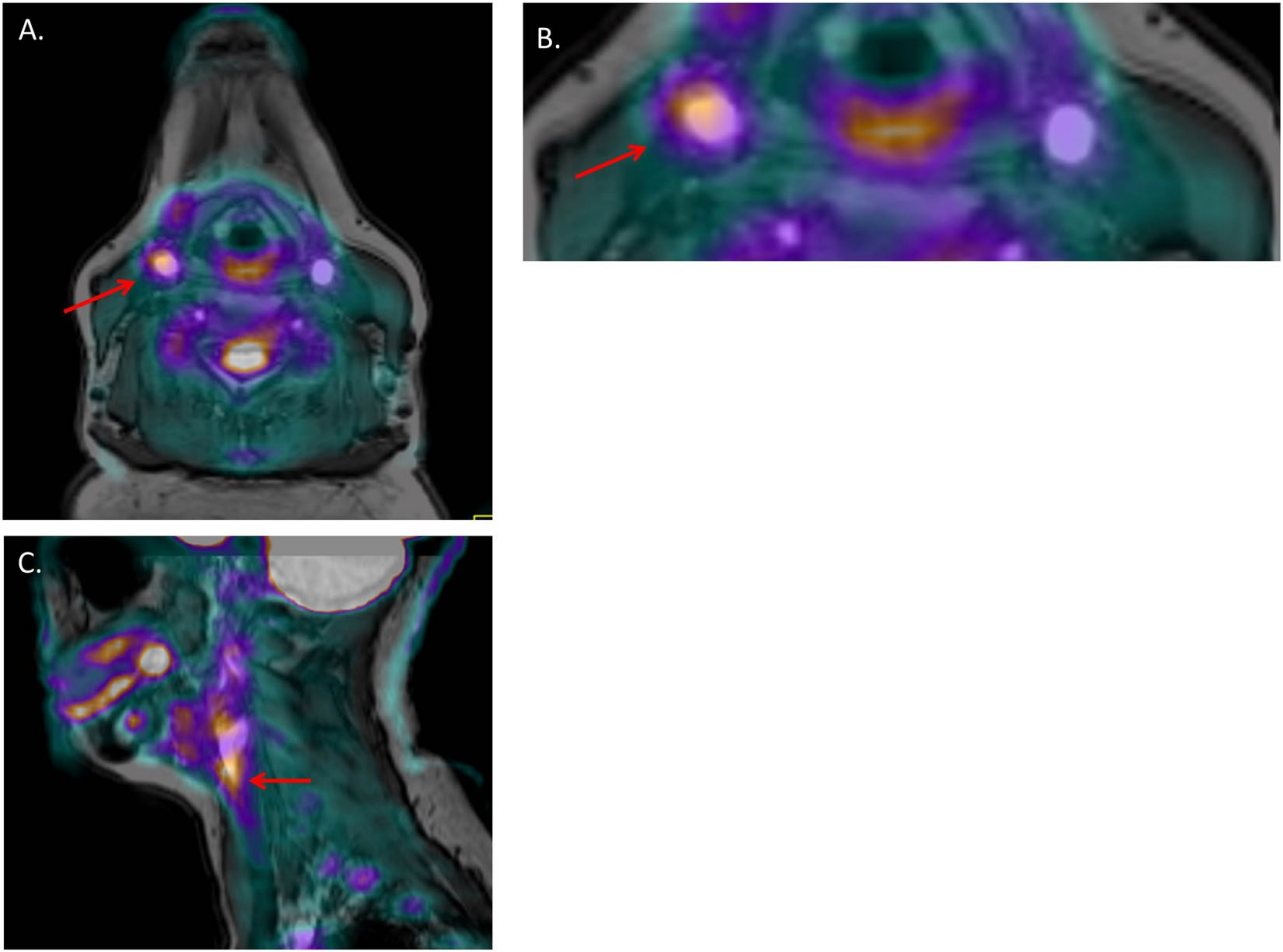
Provides an example of a carotid FDG-PET-MRI in an RA patient. (**A**) A cross sectional image at the level of the plaque. (**B**) Provides a more magnified image of the plaque on this slice which demonstrates increased uptake around the vessel wall where plaque has been identified (highlighted by the red arrow). (**C**) A coronal section in the same patient with the red arrow highlighting the area of increased uptake within the plaque in this view.

13 RA patients underwent carotid artery PET-CT. In all cases, registration was possible with MRI T_1_- weighted images and representative example images are presented in Fig. 4. FDG uptake was detected in all 13 cases and 12 out of 13 cases had an SUV_max_ > 1.85 (the proposed threshold for carotid wall inflammation^34^). The median (IQR) SUV_max_ of plaque was 2.18 (2.00, 2.65) and SUV_max_ correlated significantly with hs-CRP (r = 0.58, *p* = 0.04). There was no association with plaque volume, *K* ^trans^, IL6 levels or DAS28 score (r = 0.20[*p* = 0.609]; r = 0.14[*p* = 0.74]; r = 0.13[*p* = 0.697]; r = −0.15[*p* = 0.617], respectively).

Median (IQR) SUV_max_ in non-atheromatous artery was 2.23(1.62, 2.56). There was a trend towards a correlation between SUV_max_ in atheromatous and non-atheromatous wall (r = 0.42, *p* = 0.078). 5/13 subjects had more than 30% higher SUV_max_ values in plaque than in non-atheromatous wall, suggesting preferential plaque inflammation in these cases.

## Discussion

This study adds to the literature demonstrating higher prevalence of atherosclerosis in RA and an association between plaque and both traditional risk factors and inflammation. The association of hs-CRP with plaque presence and inflammation and the independent association of IL6 with plaque accords with the theory of the IL6/CRP axis being a significant driver of cardiovascular risk in the general population and the hypothesis that excessive inflammation contributes to increased risk in RA^15^.

### MRI findings

Despite there being no significant difference in plaque size, an increased prevalence of carotid plaque calcification was noted in patients. Previous published data suggests that 14 subjects are required per group to compare plaque composition on MRI^35^. Although the control group was small, the presence of calcification in the RA group (n = 15) was also higher than described in studies of asymptomatic individuals in the literature. Underhill *et al*. evaluated carotid plaque composition in 191 patients with asymptomatic carotid stenosis, comparing findings in those with and without significant coronary artery disease. The mean age of participants included in this study ranged from 57.8 and 60.5 years (in male without coronary disease and female with coronary disease respectively), similar to the age of patients in the current study. Underhill *et al*. demonstrated a prevalence of calcification of 40.2% and 16% in patients with and without coronary disease respectively^36^. We found plaque calcification in 70% of patients with RA, only one of whom had a history of stable angina. Carotid plaque calcification is a predictor of subsequent stroke and more severe coronary disease^37, 38^. Although calcification is a sign of advanced plaque, calcium deposition can occur early, often in response to tissue necrosis or cholesterol deposition. Pro-inflammatory cytokines, in particular TNF, are known to upregulate signalling pathways promoting vascular calcification, and higher levels of pro-inflammatory cytokines have been found preferentially in areas of calcified plaque^39, 40^. In RA increased vascular calcification has been noted and found to be associated with CRP levels^41^. However to date, measurement of vascular calcification has been thought to reflect an overall increase in atherosclerosis burden in RA. Although the size of the control group is a major limitation in the current study, when compared with the literature, our data provides preliminary evidence to support early plaque calcification in RA and may suggest an altered natural history of plaque formation and progression. This highlights the need for further investigation into the role of chronic inflammation on vascular calcification in RA.

We found no difference in DCE measurements of plaque inflammation and neovascularisation between groups. There are a number of factors that may contribute to the negative finding. The first is the small sample size. At the time of study set-up, carotid DCE-MRI was a novel technique and little was known about the sample size required to power a study comparing DCE parameters. However, a study published in 2014 suggested that 50 MRI datasets per group were required to achieve statistical power^42^. The increased prevalence of calcification in the RA group may also have confounded measurements of *K* ^trans^. Calcium nodules are avascular, hence no contrast would enter this area of plaque which in turn could lead to a lower measured *K* ^trans^ in plaques with calcium. We noted a trend towards an inverse correlation between calcium content and *K* ^trans^ measurement, which accords with previous studies^27, 43^. Thus, higher calcium content of plaque is likely to have contributed to lower overall *K* ^trans^ values in the RA cohort in this study. Development of methods to exclude areas of calcification from the region of interest on DCE-MRI would go some way to address this issue and may be an area for further research.

A number of MRI scans (16/36) were not suitable for analysis mainly because the plaque thickness was <2 mm on MRI, despite reaching this threshold on US. US is operator-dependent even with expert sonographers. We also noted that in some cases, what appeared as plaque on US, was actually an area of minimal thickening at the origin of a small collateral branch off the carotid bulb on MRI. This may have led to an overestimation of plaque thickness on US, a phenomenon not well described in the literature. No false positive or negative rates are available for US and MRI. However, in a study where MRI scans were performed in patients with plaque causing >15% stenosis on US, only 31/123 cases had plaque >2 mm thick^44^. Imaging small plaques can be technically challenging and evidence suggests the inclusion of plaques causing more than 30% stenosis or measuring plaque thickness on MRI at inclusion, may be a more reliable way of identifying plaque suitable for DCE-MRI analysis in future studies^42, 45^. While DCE-MRI appears to be an effective method of evaluating plaque inflammation in stroke patients, the large numbers that would need to be screened in order to obtain a sample size of 50 subjects with asymptomatic plaques may be a limiting factor when considering implementation of carotid DCE-MRI in larger RA studies in the future. However, only 14 subjects per group are required to power a study for compositional analysis of plaque, thus MRI is still a valuable and feasible option for the study of other high risk features of plaque in future studies^34^. We note the technical challenges of carotid MRI and that in any future study using these methods in an RA population, these feasibility factors should be considered.

### PET findings

PET-MRI analysis was performed in 13 patients and a degree of FDG uptake was detected in all cases. Although normal values for FDG uptake within the carotid artery are not well established, a recent paper by Van Der Valk *et al*. proposed a threshold of SUVmax > 1.85 for significant carotid artery inflammation^34^. This value was proposed as it was above the 90th percentile in healthy volunteers who had no clinical cardiovascular disease, nor any significant risk factors. Other studies in Takayasu's arteritis have also suggested that an SUV max >1.3 signifies vascular inflammation^46^. If we apply the more conservative thresholds to our data 12/13 patients would be deemed to have significant inflammation. However we acknowledge that there were differences in acquisition and reconstruction methods between Van der Valk’s study and our own, so these may not be completely comparable data. Although no control group was included in the current study, in the published literature the prevalence of plaque inflammation in asymptomatic subjects is estimated to be 29% thus the current data would support the hypothesis that patients with RA may have more inflammatory plaques^47^.

Maki-Petaja *et al*. also demonstrated that patients with active RA had aortic inflammation on FDG-PET and that vascular inflammation improved following anti-TNF therapy^8^. Our data showing FDG uptake in non-atheromatous walls in RA patients, supports the hypothesis that RA patients have generalised arterial inflammation. However, in 5 cases we found a >30% higher SUV_max_ in plaque than in non-atheromatous wall, suggesting that plaque inflammation, is not simply a reflection of generalised wall inflammation, but a biomarker of an active inflammatory process that may result in plaque vulnerability and an enhanced risk of a future CVE. The significant correlation between CRP and FDG uptake supports the hypothesis that systemic inflammation not only influences plaque development but also plaque stability. Other studies have also reported an association between CRP and atherosclerosis, as well as future cardiovascular events in RA (48, 49). Our study adds to this literature by demonstrating a link between systemic and local plaque inflammation in RA and may explain why atherosclerotic lesions in RA are more vulnerable to early rupture.

PET-CT was well tolerated by patients and co-registration was possible with MRI sequences in all cases, suggesting PET may be an effective method of quantifying plaque inflammation in future RA studies.

### Limitations

We acknowledge several limitations to the study, including the limited power to detect differences in MRI measurements. We do note however that it provides valuable feasibility data with which to power future studies in this population. Additionally, subjects on statins were excluded from the study, as these agents influence MRI and PET measurements. This likely led to exclusion of patients at particularly high risk of CVD which whilst limiting generalisability would, if anything, bias us towards observing fewer differences between patients and controls.

Although the groups were age- and sex-matched, there was a higher prevalence of some traditional risk factors in the patient group, which meant that traditional risk factors may have contributed to the differences in plaque prevalence and phenotype observed on MRI. However the association between a number of measures of inflammation including joint disease activity and IL6 levels remained significant on adjustment for traditional risk factors. This emphasises the importance of disease specific factors in the development of atherosclerosis in RA. Additionally the independent association between IL6 and plaque in patients strongly suggests that inflammation is playing an important role in cardiovascular risk in RA.

We did not recruit a control group for the PET sub-study as the radiation exposure could not be justified in the context of a pilot study. PET and MRI were performed on different scanners and images were co-registered. While images were co-registered satisfactorily in all cases, small anatomical discrepancies due to differences in neck flexion could not be completely excluded and therefore, potentially may have contributed to a measurement error of SUV_max_. This has been an inherent problem in the past, with PET-MRI studies being performed on separate scanners, and not restricted to the current study.

In conclusion, we have shown an increased prevalence of atherosclerosis which was associated with classical cardiovascular risk factors and inflammation. On MRI we noted plaque calcification in RA patients, providing preliminary evidence to support earlier calcification and a more high risk plaque phenotype in this patient group. MRI and FDG-PET are well tolerated, feasible techniques to investigate plaque morphology and inflammation. However careful power and sample calculations are needed due to the technical demands involved. MRI and PET may provide the opportunity to interrogate the natural history of plaque and elucidate the influence of disease activity and anti-inflammatory therapies on atherosclerosis in RA. Additionally the development of combined PET-MRI scanners may allow a comprehensive assessment of morphology and inflammation of plaque in one imaging session which may be more feasible in patients with inflammatory joint diseases.

## Electronic supplementary material

Supplementary data
