# Supplementary material for: Imaging atherosclerosis in rheumatoid arthritis: evidence for increased prevalence, altered phenotype and a link between systemic and localised plaque inflammation
Source: Sci Rep. 2017 Apr 11;7:827. doi: 10.1038/s41598-017-00989-w (PMC5429790; doi:10.1038/s41598-017-00989-w)
Supplement: Supplementary file 1 — Supplementary data [file 41598_2017_989_MOESM1_ESM.doc]

**Imaging atherosclerosis in rheumatoid arthritis: evidence for increased prevalence, altered phenotype and link between systemic and localised plaque inflammation**

**S. Skeoch^1,2^, P. L. Hubbard Cristinacce^3,4^, H. Williams^5^, P. Pemberton^6^, D. Xu^7^, S. Jie^7^, J. James^5^, C. Yuan^7^, T. Hatsukami^7^, P.D. Hockings^8,9^, M.Y. Alexander^10^, J.C. Waterton^3^, I.N. Bruce^1,2^**

^1^ Arthritis Research UK Centre for Epidemiology, Centre for Musculoskeletal Research, Faculty of Medicine, Biology and Health, The University of Manchester, Manchester Academic Health Science Centre, Manchester, United Kingdom.

^2^ The Kellgren Centre for Rheumatology, NIHR Manchester Musculoskeletal Biomedical Research Unit, Central Manchester University Hospitals NHS Foundation Trust, Manchester Academic Health Science Centre, Manchester, United Kingdom.

^3^Centre for Imaging Sciences, The University of Manchester, Manchester Academic Health Science Centre, Manchester, United Kingdom.

^4^School of Psychological Sciences, The University of Manchester, Manchester, United Kingdom.

^5^Department of Nuclear Medicine, Central Manchester University Hospitals NHS Foundation Trust, Manchester Academic Health Science Centre, Manchester, United Kingdom.

^6^Specialist Assay Laboratory, Central Manchester University Hospitals NHS Foundation Trust, Manchester Academic Health Science Centre, Manchester, United Kingdom.

^7^Department of Radiology, University of Washington, Seattle, United States of America.

^8^MedTech West, Chalmers University of Technology, Gothenberg, Sweden.

^9^Antaros Medical, Mölndal, Sweden.

^10^Healthcare Science Research Institute, Manchester Metropolitan University, Manchester, UK

***Supplementary data***

**MRI protocols**

Equipment specification

Scans were performed on a 3 T Philips Achieva (Philips Healthcare, Best, The Netherlands) using an 8 channel phase array surface coil specifically designed for carotid artery imaging (Shanghai Chenguang Medical Technologies Co. Ltd , China). Supporting software was provided The Department of Radiology, University of Washington.

Imaging acquisition

A previously validated imaging protocol was used and details of imaging sequences can be seen in Table 1 (23, 24). Patients lay in the scanner with a fixed position headrest with head position aligned with the long axis of the scanner. A measurement from chin to mandible was made at this point to optimise co-registration between PET and MRI images. An initial survey then time of flight sequence was acquired then subsequent sequences were centred round the bifurcation of the index artery.

Table 1. MRI acquisition sequences

| **Sequence** | **TR (ms)** | **TE (ms)** | **Flip angle (°)** | **Slice thickness (mm)** | **Number of slices** | **Matrix** | **Voxel dimensions (mm)** |
| --- | --- | --- | --- | --- | --- | --- | --- |
| **Survey** | 6.2 | 2.38 | 15 | 4 | 33 | 256 × 256 | 0.9375 x 0.9375 x 4 |
| **2D-TOF** | 25.15 | 9.93 | 40 | 2 | 36 | 256 × 256 | 0.625 x 0.625 x 0.625 |
| **Sagittal Oblique** | 2000 | 7.50 | 90 | 2 | 6 | 256×256 | 0.3125 x 0.3125 x 0.3125 |
| **Proton density weighted TSE** | 4800 | 50 | 90 | 2 | 16 | 268 × 268 | 0.2857 x 0.2857 x 2 |
| **T_2_weighted TSE with MDIR** | 4800 | 50 | 90 | 2 | 16 | 268 × 268 | 0.2857 x 0.2857 x 2 |
| **3D-TOF** | 20 | 4.88 | 20 | 2 | 48 | 268 × 268 | 0.2857 x 0.2857 x 2 |
| **MP-RAGE** | 8.68 | 5.24 | 15 | 2 | 48 | 268 × 268 | 0.2857 x 0.2857 x 2 |
| **T_1_ weighted 3D TFE*** | 8.40 | 3.90 | 8 | 2 | 156 | 224 × 224 | 1 x 1 x 2 |
| **T_1_ weighted TSE with QIR** | 800 | 10 | 90 | 2 | 16 | 268 × 268 | 0.2857 x 0.2857 x 2 |
| **DCE T_1_ weighted FFE** | 126 | 4.61 | 50 | 3 | 4 | 260 × 260 | 0.3125 x 0.3125 x 3 |
| **Post contrast T_1_ weighted TSE with QIR** | 800 | 10 | 90 | 2 | 16 | 268 × 268 | 0.8275 x 0.8275 x 2 |

Abbreviations: **TOF**, time of flight; **TSE**, turbo spin echo; **MP-RAGE**, magnetisation prepared rapid acquisition gradient echo; **MDIR**, multi-slice double inversion recovery; **TFE,** turbo field echo; **QIR**, quadruple inversion recovery; **DCE,** dynamic contrast enhanced; **FFE**, fast field echo. *Imaging sequence for co-registration with PET-CT

*DCE sequence*

The DCE sequence was planned from viewing the T_1_ images from the preceding sequence. 4 slices were positioned to include the carotid bifurcation and plaque in the index artery. A series of frames were taken with the imaging parameters seen in Table 1. 3 frames were taken prior to injection, then 15 successive frames during and after injection of gadopentate dimeglumine (Magnevist, Bayer HealthCare Pharmaceuticals), concentration 0.05mmol/kg, injection rate: 1cc/s. Duration of each frame was 17.5s. Data was anonymised and transferred over a secure server to the Department of Radiology, University of Washington. Readers were blinded to clinical information and case- control status. Analysis was performed as per protocols referenced in the main paper (29,30).

**^18F^FDG-PET-MRI**

Equipment specification

**Scanner:** The PET-CT scanner used in the study is the Siemens Biograph mCT·64·S. Key features of this scanner include an additional detector ring which provides extended axial coverage (21.6cm), the acquisition of time of flight (TOF) information during imaging and resolution modelling. Using TOF and resolution modelling, image contrast, signal to noise ratio and spatial resolution are improved compared to scanners without this functionality.

**Headrest**: A bespoke foam headrest was made to match head and neck support provided by the carotid coil, to enable patient positioning in the MRI scanner to be reproduced.

Imaging acquisition

Patients attended the department having fasted 6 hours prior to arrival. Height and weight was measured then blood glucose was checked using a glucometer. Providing the fasting glucose was less than 10mmol/L, a cannula was then inserted and the patient fitted with a soft cervical collar. 200MBq of FDG was injected, and the patients rested in a quiet room in a comfortable armchair, for 2 hours.

Following the rest, subjects were asked to lie on the PET scanner bed. The headrest was placed to support the head and neck in the same manner as the carotid coil, and lasers used to check the head was aligned with the long axis of the scanner and the coronal plane through the middle of the neck was centred in the trans-axial field of view. Chin to sternal notch distance was measured and if it was different from the measurement taken during the MRI scan then the patient would be repositioned to ensure the measurements matched.

Images were acquired over a 20 minute period. First a lateral CT topogram was acquired from top of ears to sternal notch. The field of view for PET-CT was defined so that it was centred on the known plaque location from ultrasound; if plaque was extensive then a point was chosen midway between the known axial locations of both carotid bifurcations. CT for semi-localisation and attenuation was acquired, followed by a 1 bed PET acquisition in list-mode which is where every pair of photons captured were logged as they come in with a time stamp. This allows for greatest flexibility in image reconstruction and analysis following acquisition.

Image processing

Images were reconstructed using the Siemens UHD (ultra-high definition) reconstruction algorithm, which incorporates the TOF data and corrects for the variable special resolution within the field of view. Reconstruction using Siemens UHD was performed on the scanner or on a clinical work station running the same software, using 3 iterations in 24 subsets, a 400×400 matrix and a 1mm Gaussian post filter.

Co-registration of reconstructed images with the T_1_ weighted MRI sequence was then performed on Siemens Syngo clinical work station using TrueD software. The CT and MRI were registered automatically using a rigid transform generated on the basis of mutual information. Where local landmarks were visible, further manual rigid translation was applied to register the CT to the MRI. In most cases, the only reliable local landmarks were calcifications within the plaque, which appear white on CT and black on T_1_ weighted MRI. The final transform was then applied to register PET and MRI.

Imaging analysis

*Analysis of FDG uptake within plaque:* Plaques were identified on the T_1_ weighted MRI images and regions of interest (ROIs) were drawn around the outer surface of the artery in each slice where plaque was apparent, to define a volume of interest (VOI). The artery, rather than the plaque, was outlined to isolate the relevant structure whilst allowing for any slight mis-registration between the PET and MRI. The VOI was then applied to the fused PET-MRI images and a maximum standardised uptake value (SUV^max^) obtained for the corresponding area on the PET.

*Analysis of FDG uptake within non-atheromatous wall:* A second VOI was defined on the T_1_ weighted MRI images around a distal section of the same artery which had no significant wall thickening suggestive of plaque. This second VOI incorporated the same number of slices used to evaluate FDG uptake within plaque, centred around a distal section of the same artery, which had no significant thickening. The VOI was then applied to the fused PET-MRI images and a maximum standardised uptake value (SUV^max^) obtained for the corresponding area on the PET.

Scans were analysed by HW, Senior Medical Physicist who was blinded to DCE MRI findings, inflammatory markers results and RA disease activity but was aware of a limited medical history to ensure that patients were suitable and positioned appropriately for PET-CT imaging.
